# The Identification of RNA-Binding Proteins Functionally Associated with Tumor Progression in Gastrointestinal Cancer

**DOI:** 10.3390/cancers13133165

**Published:** 2021-06-24

**Authors:** Hiroaki Konishi, Shin Kashima, Takuma Goto, Katsuyoshi Ando, Aki Sakatani, Hiroki Tanaka, Nobuhiro Ueno, Kentaro Moriichi, Toshikatsu Okumura, Mikihiro Fujiya

**Affiliations:** 1 Department of Gastroenterology and Advanced Medical Sciences, Asahikawa Medical University, 2-1-1-1, Midorigaoka, Asahikawa 078-8510, Japan; hkonishi@asahikawa-med.ac.jp; 2 Division of Metabolism and Biosystemic Science, Gastroenterology, and Hematology/Oncology, Department of Medicine, Asahikawa Medical University, Asahikawa 078-8510, Japan; shin1014@asahikawa-med.ac.jp (S.K.); t-gotti@asahikawa-med.ac.jp (T.G.); k-ando@asahikawa-med.ac.jp (K.A.); sakatani@asahikawa-med.ac.jp (A.S.); u-eno@asahikawa-med.ac.jp (N.U.); morimori@asahikawa-med.ac.jp (K.M.); okumurat@asahikawa-med.ac.jp (T.O.); 3 Division of Tumor Pathology, Department of Pathology, Asahikawa Medical University, Asahikawa 078-8510, Japan; hiroki-t@asahikawa-med.ac.jp

**Keywords:** RBP, gastrointestinal cancer, functional assessment, expressional changes, genetic mutation

## Abstract

**Simple Summary:**

Previous investigations described bioinformatic analyses based on the mRNA expression and somatic mutation as useful strategies for identifying cancer-associated molecules that were potential candidates of therapeutic targets. However, these data included secondary changes and non-functional alterations that do not influence tumor progression. Investigations, including our own studies, have shown that some RBPs shuttle cytoplasm and nuclei, and their affinity to RNAs is regulated by posttranslational modifications, such as phosphorylation. Therefore, the functional assessment of individual molecules is the most suitable strategy for identifying cancer-associated genes with or without expressional changes and mutations. This report showed for the first time that a functional assessment using an siRNA library was useful for identifying therapeutic targets from molecular groups, including RBPs, that had not been identified by expressional and mutational analyses.

**Abstract:**

Previous investigations have indicated that RNA-binding proteins (RBPs) are key molecules for the development of organs, differentiation, cell growth and apoptosis in cancer cells as well as normal cells. A bioinformatics analysis based on the mRNA expression and a somatic mutational database revealed the association between aberrant expression/mutations of RBPs and cancer progression. However, this method failed to detect functional alterations in RBPs without changes in the expression, thus leading to false negatives. To identify major tumor-associated RBPs, we constructed an siRNA library based on the database of RBPs and assessed the influence on the growth of colorectal, pancreatic and esophageal cancer cells. A comprehensive analysis of siRNA functional screening findings using 1198 siRNAs targeting 416 RBPs identified 41 RBPs in which 50% inhibition of cell growth was observed in cancer cells. Among these RBPs, 12 showed no change in the mRNA expression and no growth suppression in non-cancerous cells when downregulated by specific siRNAs. We herein report for the first time cancer-promotive RBPs identified by a novel functional assessment using an siRNA library of RBPs combined with expressional and mutational analyses.

## 1. Introduction

RNA regulation, including the transportation, splicing, polyadenylation, degradation, stabilization and translation of mRNA, non-coding RNA and microRNA, is essential for maintaining homeostasis and associated with the development and differentiation of tissues and organs [1,2]. RNA-binding proteins (RBPs), which have RNA recognition motifs, are closely associated with RNA regulation, and a total of 424 human RBPs, 413 mouse RBPs, 257 fly RBPs and 244 worm RBPs were registered to an RBP database (RBPDB) (http://rbpdb.ccbr.utoronto.ca, accessed on 3 February 2021). Several RBPs are evolutionally conserved and ubiquitously expressed because of their essential roles in cellular events, such as cell growth, apoptosis and senescence [3,4].

Previous investigations have shown that some RBPs, such as heterogeneous ribonucleoproteins (hnRNPs) and Musashis, are abnormally expressed in several cancer cells, including colorectal, pancreatic and breast cancer, and promote tumor cell progression via the inhibition of apoptosis and acceleration of cell growth [5,6]. Importantly, the expressional changes of some RBPs, including HuR (ELAVL1) and PTBP1, are aberrantly expressed and co-related with the prognosis of ovarian and hepatocellular carcinoma patients [7,8]. Likewise, Lin28B, which is highly induced in heterogeneous circulating tumor cells of pancreatic ductal adenocarcinoma (PDAC) patients, may be a therapeutic target of metastasis as well as a prognostic marker [9]. These findings suggest that the aberrant expression of RBPs is closely associated with cancer progression.

With the development of bioinformatics technologies, the expressional and mutational abnormalities of malignant lesions have been exhaustively assessed using metadata registered to in silico databases, such as the Gene Expression Omnibus (GEO) and the Cancer Genome Atlas (TCGA) for expressional abnormalities and the Catalogue of Somatic Mutations in Cancer (COSMIC) for mutational abnormalities. This has facilitated the establishment of novel therapeutic targeted molecules and prognostic marker molecules, including RBPs [10].

However, cancer cells have been shown to frequently utilize post-translational regulation rather than expressional significance for their progression and survival. Our previous studies revealed that some RBPs, such as hnRNP A0 and hnRNP A1, have oncogenic properties, including anti-apoptotic functions and the promotion of cell growth, and their oncogenic functions are regulated by protein modification, including phosphorylation and ubiquitination, rather than expressional significance [11,12]. Importantly, the mRNAs that bind to hnRNP A0 differ completely depending on the phosphorylation status of hnRNP A0. For example, the cancer-progressive function of hnRNP A0 was exhibited only when it was phosphorylated. A previous report showed that hnRNP A1 localized to the cytoplasm in multiple myeloma cells only when phosphorylated, resulting in the promotion of cell growth through the translation of cancer-related mRNAs, including MYC [13]. Likewise, the degradation of hnRNP A1 and binding between hnRNP A1 and tumor-associated mRNAs, such as CDK6, were modified by the microRNA binding status in colorectal cancer cells [11,14]. These findings indicate that changes in the protein expression as well as functional assessments need to be considered in order to identify cancer therapeutic targets. 

In this study, we constructed an siRNA library based on the RBPDB and performed a functional assay while comparing the gene expression profiles and a mutational database of digestive cancer cells to identify tumor-promotive RBPs without gene mutations or without overexpression in order to investigate novel therapeutic targets for digestive cancer.

## 2. Materials and Methods

### 2.1. Cell Culture

Human cancer cell lines were grown in McCoy’s 5A Medium (HCT116 (ATCC, Manassas, VA, USA)), Roswell Park Memorial Institute (RPMI) 1640 (SW480 (ATCC), PANC-1 (ATCC), OE33 (DS Pharma Biomedical Co., Ltd., Osaka, Japan), KYSE70 (DS Pharma Biomedical Co., Ltd., Osaka, Japan)) or high-glucose Dulbecco’s Modified Eagle’s Medium (DMEM) (SUIT-2 (Health Science Research Resources Bank, Osaka, Japan)) supplemented with 10% (*vol*/*vol*) fetal bovine serum (FBS), 2 mM l-glutamine, 50 U/mL penicillin and 50 µg/mL streptomycin in a humidified atmosphere containing 5% CO_2_. Human primary pancreatic endothelial cells (HPPECs; Cell Biologics Inc., Chicago, IL, USA), Het1As (non-tumorous esophagus cells; ATCC) and HCEC-1CTs (Summit Pharmaceuticals International Corporation, Tokyo, Japan) were grown in Epithelial Cell Medium (Cat#H6621; Cell Biologics), Bronchial Epithelial Cell Growth Basal Medium (Lonza, Basel, Switzerland) and ColoUp medium (DMEM/Medium 199 Earle’s, 4 + 1; Cat# F0435 and Cat# FG0615; Biochrom Ltd., Cambridge, UK) containing 4 mM GlutaMAXTM-1 (100X), (Cat# 35050-038; Thermo Fisher Scientific, Waltham, MA, USA) 2% Cosmic Calf Serum (Cat# SH30087; Hyclone Laboratories Inc., Logan, UT, USA), 20 ng/mL EGF (Cat# E9644; Sigma-Aldrich Co., LLC, St. Louis, MO, USA), 10 μg/mL Insulin (Cat# I9278; Sigma Aldrich), 2 μg/mL Apo-Transferrin (Cat# T2036; Sigma Aldrich), 5 nM Sodium-Selenite (Cat# S5261; Sigma Aldrich) and 1 μg/mL Hydrocortisone (Cat# H0396; Sigma Aldrich). The characteristics of each cell line are shown in Table 1, and the cancer-specific functions were assessed by a comparison with non-cancerous cells from each organ.

### 2.2. siRNA and Transfection

The list of 424 human RBPs was downloaded from the RBPDB. A total of 1192 siRNAs targeting 416 RBPs (siRNAs that effectively downregulate 8 RBPs could not be established in this study) were selected from Silencer Select siRNA Libraries (Thermo Fisher Scientific). Transfection was performed using Lipofectamine RNAiMAX (Thermo Fisher Scientific) in triplicate.

### 2.3. SRB Assays

Cells were first seeded on 96-well microplates at 0.75 × 10^4^ cells per well. The cells were then fixed in 5% trichloroacetic acid (TCA) for 1 h at 4 °C and washed 4 times in distilled water. The microplates were then dehydrated at room temperature, stained in 100 μL/well of 0.057% (wt/vol) SRB powder/distilled water, washed 4 times in 0.1% acetic acid and re-dehydrated at room temperature. The stained cells were lysed in 10 mM Tris-buffer, and the optical density (OD) was measured at 510 nm.

### 2.4. MTT Assays

The cells were seeded on 96-well microplates at 0.5–0.75 × 10^4^ per well. Cell growth was assessed using an MTT cell proliferation kit according to the manufacturer’s instructions (Roche Applied Science, Indianapolis, IN, USA). The OD was measured at a 590 nm test wavelength and a 620 nm reference wavelength.

### 2.5. Western Blotting

Total proteins were extracted from samples using NP-40 Cell Lysis buffer containing protease inhibitor and phosphatase inhibitor. Equal amounts of protein were resolved using sodium dodecyl sulfate–polyacrylamide gel electrophoresis (SDS-PAGE) (12.5%), blotted to a nitrocellulose membrane and blocked in SuperBlock™ (TBS) Blocking Buffer (ThermoFisher Scientific, Carlsbad, CA, USA). Blots were incubated overnight at 4 °C with primary antibodies of Cyclin B1 (Abcam, Cambridge, UK), Cyclin D1 (Abcam) or PARP (Cell Signaling Technologies, Danvers, MA, USA). The blots were washed in TBS containing 0.05% Tween 20 (T-TBS), incubated with HRP-conjugated secondary antibodies (R&D Systems, Minneapolis, MN, USA), washed in T-TBS, and then developed using either the Super-Signal West Pico or the femto enhanced chemiluminescence system (ThermoFisher Scientific). The averaged protein expression was normalized to the actin expression (BD Transduction Laboratories, Lexington, KY, USA). Detailed information can be found at Supplementary Figure S2.

### 2.6. cDNA Analyses

Total RNA was extracted using an RNeasy mini kit (Qiagen, Venlo, The Netherlands) according to the manufacturer’s instructions. The mRNA profiling was investigated using the Clariom S array (Thermo Fisher Scientific). A ≥2-fold difference was considered to indicate a significant change.

### 2.7. Data Corrections

The growth inhibition effect of each cell was calculated by the following formula using Microsoft Excel: growth inhibition rate (%) = [1 − (OD510 nm at day 3 of siRNA of each RBP-OD510 nm at day 1 of siRNA of each RBP)/(OD510 nm at day 3 of Scrambled RNA − OD510 nm at day 1 of Scrambled RNA)] × 100. In this study, RBPs that showed a growth inhibition rate of >50% when siRNA was transfected were defined as tumor-promotive RBPs. The raw data of the cDNA array were analyzed using the Transcriptome Analysis Console (ThermoFisher Scientific). The overlaps of genes were detected using Microsoft Excel. The graphs of each experiment and Venn diagram were generated using the Microsoft Excel and PowerPoint software programs. The densitometry of Western blots was analyzed using the Image J software program. The interactome analysis of RBPs selected by the functional assessment and cDNA array analysis was performed using the MetaCore software program (Clarivate, Philadelphia, PA, USA).

### 2.8. Statistical Assessments

The assay data were analyzed using Student’s *t*-test. *p*-values of <0.05 were considered statistically significant.

## 3. Results

### 3.1. Growth Change by the Knockdown of RBPs in Gastrointestinal Cancer Cells

To identify which RBPs are involved in tumor proliferation in gastrointestinal cancer cells, we constructed an siRNA library to perform a functional analysis of RBPs registered in the RBPDB. A total of 1198 siRNAs targeting human 416 RBPs were collected (Table S1) (siRNA targeting eight RBPs could not collected because effective sequences for siRNA were not detected in the mRNAs of these RBPs), and each siRNA was transfected to colorectal cancer cells (HCT116, SW480), pancreatic cancer cells (SUIT-2, PANC-1) and esophageal cancer cells (OE33, KYSE70). Their cell proliferation was examined by a Sulforhodamine B (SRB) assay, and RBPs with an over 50% growth reduction or a promotion effect after the transfection of siRNAs at 72 h were defined as tumor cell growth-associated RBPs in this study. The growth inhibitory effects of HCT116, SW480, SUIT-2, PANC-1, OE33 and KYSE70 were over 50% following the downregulation of 80, 3, 97, 23, 107 and 24 genes, respectively (for the growth suppression effect of each cell, see Tables S2–S8). 

### 3.2. The Association between Expressional Changes and Tumor Growth Promotive Functions of RBPs in Gastrointestinal Cancer Cells

**Figure 1:**
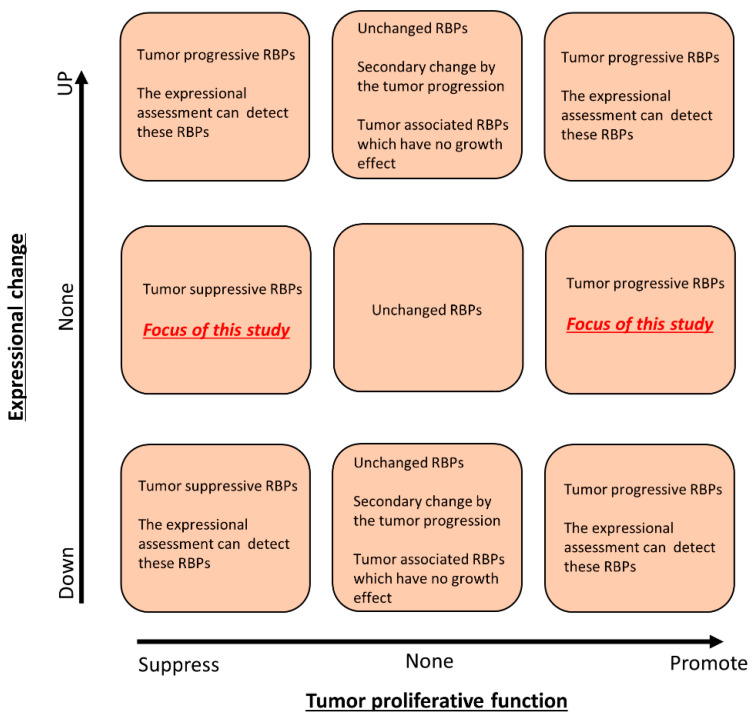
Schematic depiction of the functional and expressional screening of RBPs. A functional analysis of the siRNA library identified RBPs that promote or suppress tumor cell growth, and a gene expressional analysis using cDNA microarray identified RBPs whose expression was abnormally changed in cancer cells. In this study, we focused on the tumor proliferation-related RBPs whose expression was not changed in cancer cells compared to non-cancer cells.

A bioinformatics analysis, which highlights the expressional changes in cancer cells, reveals tumor-promotive or tumor-suppressive molecules. However, the expressional changes in cancer cells contain the secondary changes induced by cancer driver signaling, such as KRAS [15], HIF [16] and p53 [17] signaling; thus, functional assessments, such as gene silencing and gene overexpression techniques, are applied to assess whether or not an aberrantly expressed molecule may be a cancer therapeutic target. Likewise, the tumor-promotive functions of some cancer-associated molecules are regulated by posttranslational modifications, including phosphorylation and ubiquitination, rather than expression changes. Therefore, essential molecules for cancer progression will not be detected by strategies based on the expressional changes, and we hypothesized that functional RBPs, the expression of which was not changed in cancer cells, would be a novel therapeutic target for gastrointestinal cancer (for the theory and strategy of this study, see Figure 1). 

**Figure 2:**
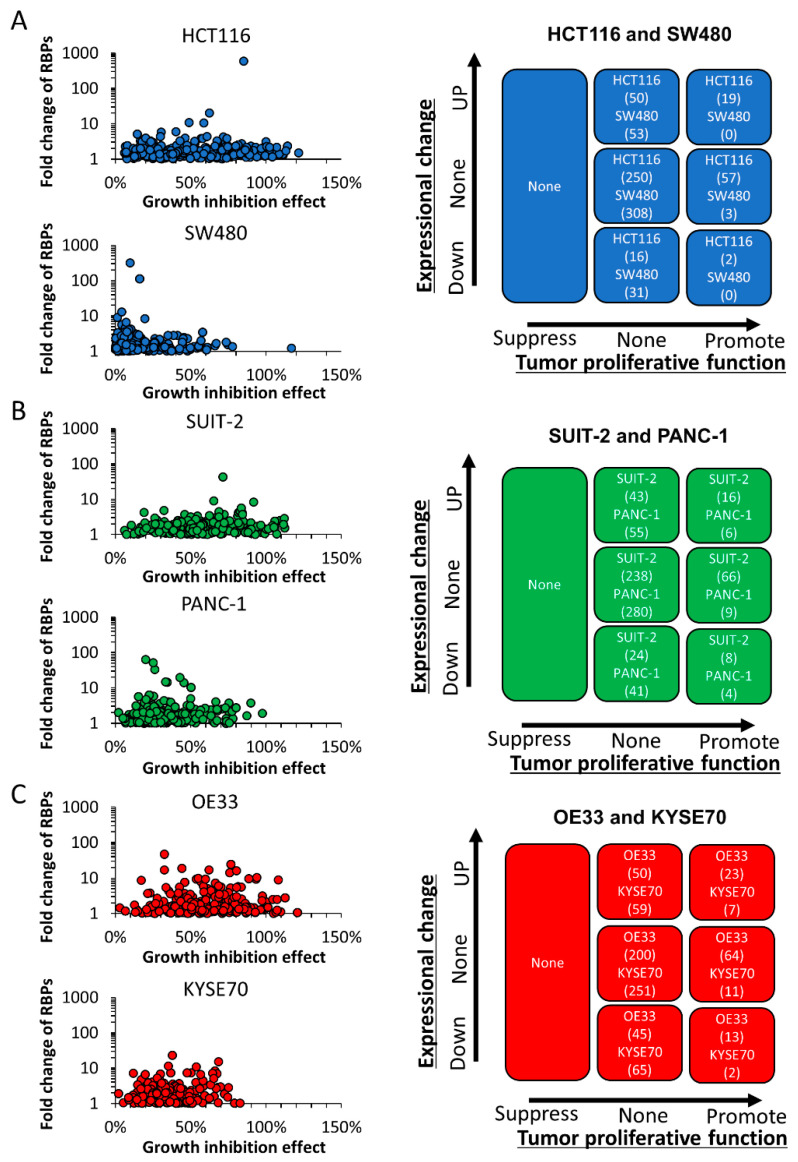
The assessment of the correlation of the functional and expressional significance in cancer cells. The correlation of the growth-promotive effect and expressional significance of RBPs was assessed by an SRB assay and cDNA microarray analysis in colorectal cancer cells (**A**), pancreatic cancer cells (**B**) and esophageal cancer cells (**C**). The number of genes is described for each cell. The tumor-proliferative function was evaluated according to the following criteria: tumor-promotive RBPs, RBPs with a >50% growth reduction effect; tumor-suppressive RBPs, RBPs with a >50% promotion effect. Likewise, the mRNA expression changes were classified according to the following criteria: upregulated RBPs, RBPs over-expressed in cancer cells (≥2-fold change); downregulated RBPs, RBPs with a decreased expression in cancer cells (≥2-fold change).

To investigate the correlation between the growth-promoting effect and expressional changes in RBPs, we compared the gene expression between cancerous and non-cancerous cell lines by a cDNA array analysis in addition to the functional analysis using the siRNA library (Figure 2). A total of 395 RBPs (29 of 424 RBPs were not detected by the cDNA array) were evaluable by the cDNA array analysis, and the fold change in cancerous cells was calculated based on the signal of the non-cancerous cell lines (RBPs that were not detected by the cDNA array analysis are listed in Table S15). The signal intensity of the HCT116 and SW480 colorectal cancer cell lines was compared to that of human colon epithelial cells (HCEC-1CTs). The signal intensity of the SUIT-2 and PANC-1 pancreatic cancer cell lines was compared to that of HPPECs. The signal intensity of the OE33 and KYSE-70 esophageal cancer cell lines was compared to that of human esophageal epithelial cells (Het-1As) (for the fold change in each line, see Tables S9–S14). A total of 69, 53, 59, 61, 73 and 66 genes were over-expressed in cancerous cells (≥two-fold change); 18, 31, 32, 45, 58 and 67 genes were under-expressed in cancerous cells (<two-fold change), and the expression of 307, 311, 304, 289, 264 and 262 genes were unchanged in the HCT116, SW480, SUIT-2, PANC-1, OE33 and KYSE70 lines, respectively. The combination analysis of an SRB assay and a cDNA array analysis revealed that 19, 0, 16, 6, 23 and 7 RBPs were correlated with a growth-promoting function and overexpression; 50, 53, 43, 55, 50 and 59 genes did not have any tumor-promoting properties despite overexpression; 250, 308, 238, 280, 200 and 251 genes did not show any marked difference in expression among cancerous and non-cancerous cells or suppress the growth of cancerous cells after siRNA transfection; and 16, 31, 24, 41, 45 and 65 genes were under-expressed in cancerous cells, while an SRB assay with the siRNA library did not show any marked change in cell growth in the HCT116, SW480, SUIT-2, PANC-1, OE33 and KYSE70 lines, respectively (Table S16). The cell growth was strongly suppressed by the siRNA transfection of 57, 3, 66, 9, 64 and 11 genes without significant changes in the expression, whereas 2, 0, 8, 4, 13 and 2 genes had tumor-promoting properties despite underexpression in the HCT116, SW480, SUIT-2, PANC-1, OE33 and KYSE70 lines, respectively. 

### 3.3. Identification of Tumor Growth-Associated RBPs without Any Significant Expressional Changes in Gastrointestinal Cancer Cells

**Figure 3:**
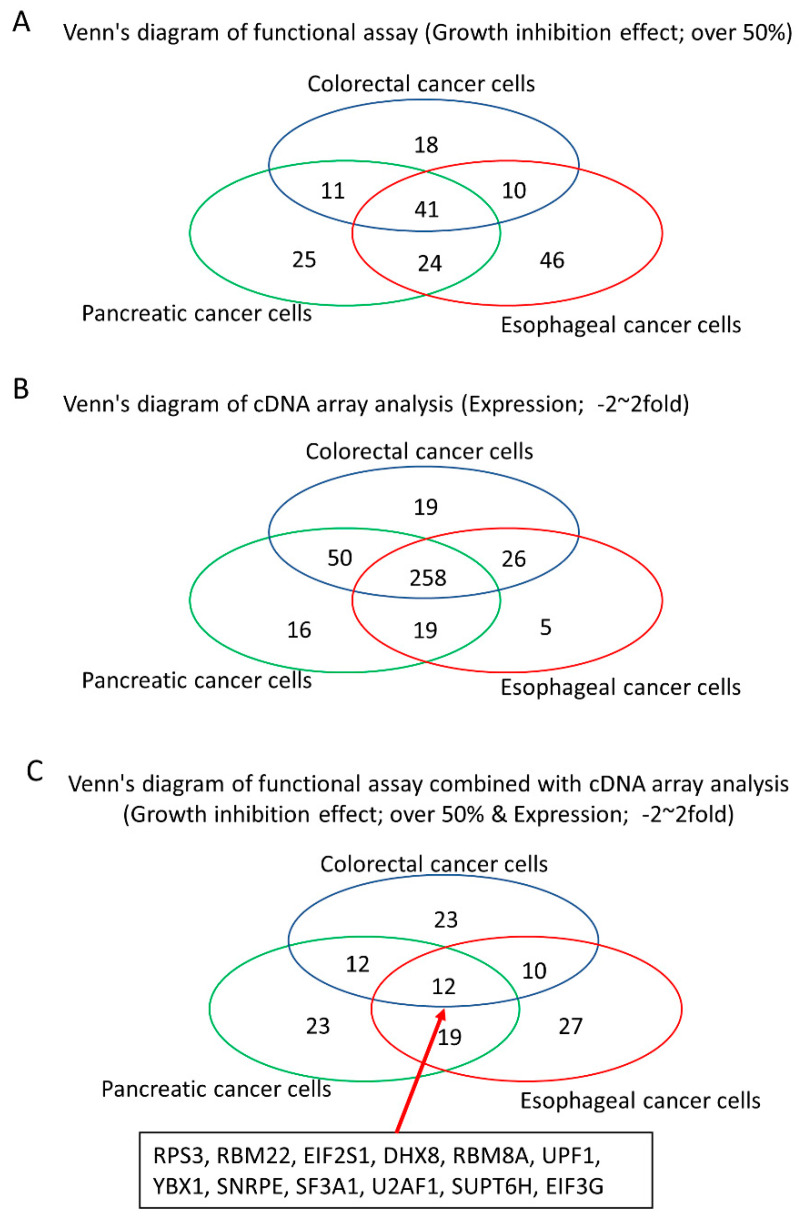
Venn diagram of the results of a functional analysis and cDNA microarray analysis. The functional (**A**) and expressional (**B**) overlap of colorectal cancer cells, pancreatic cancer cells and esophageal cancer cells is shown. Twelve RBPs were tumor-promotive RBPs without any expressional changes among cancer cell lines (**C**).

To identify the tumor driver RBPs that were unable to be detected based on the expressional data in gastrointestinal cancer cells, we assessed a Venn’s diagram of the results of the functional assay and cDNA array analysis. siRNAs of 57, 66 and 68 RBPs strongly inhibited tumor cell growth without expressional changes in colorectal cancer cells (HCT116 or SW480), pancreatic cancer cells (SUIT2 or PANC-1) and esophageal cancer cells (OE33 or KYSE70), respectively (Figure 3). Among these, 12 RBPs (RPS3, RBM22, EIF2S1, DHX8, RBM8A, UPF1, YBX1, SNRPE, SF3A1, U2AF1, SUPT6H, EIF3G) promoted tumor cell growth without expressional changes in any cancer cell line (Table 2). Strong growth inhibition (over 50%) was not detected in any non-cancerous cells (HCEC-1CT, HPPEC and Het1A) treated with the siRNA of nine RBPs (DHX8, EIF3G, RBM22, SF3A1, SNRPE, SUPT6H, U2AF1, UPF1, YBX1) (Table 3) (knockdown efficacy of these 12 RBPs shown in Table S17). 

**Figure 4:**
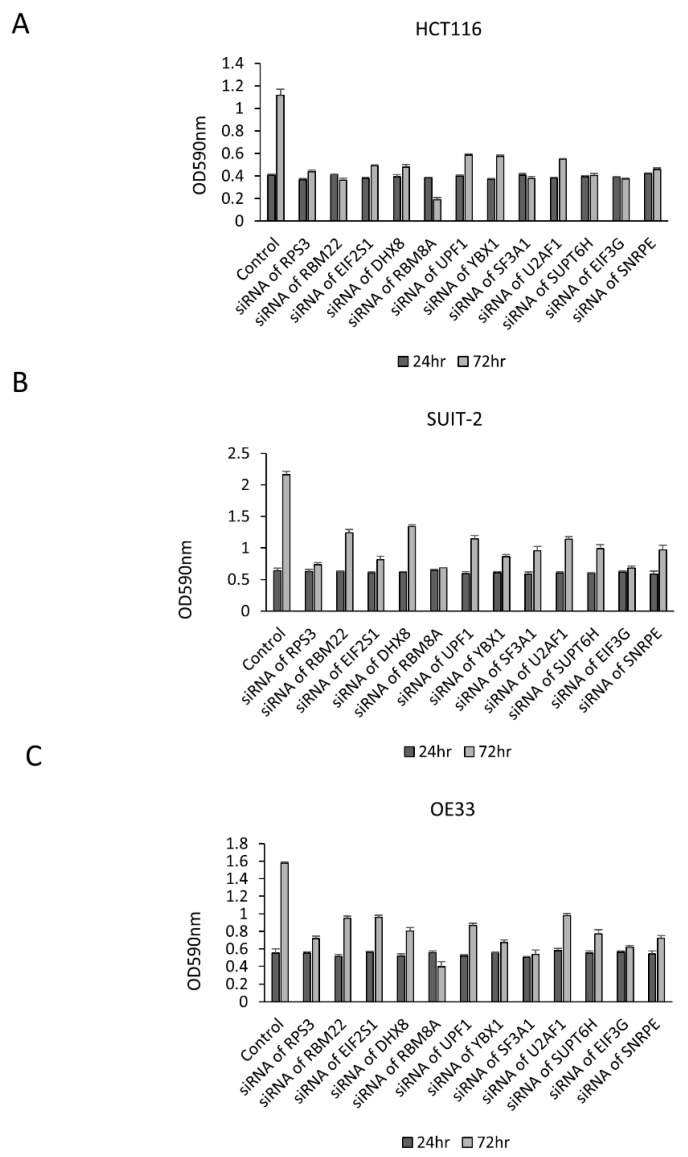
The growth changes by the downregulation of RPS3, RBM22, EIF2S1, DHX8, RBM8A, UPF1, YBX1, SNRPE, SF3A1, U2AF1, SUPT6H, EIF3G in HCT116, SUIT2 and OE33 cells. An MTT assay confirmed the growth suppression effect induced by the downregulation of cancer-related RBPs in HCT116 (**A**), SUIT2 (**B**) and OE33 (**C**) cells.

**Figure 5:**
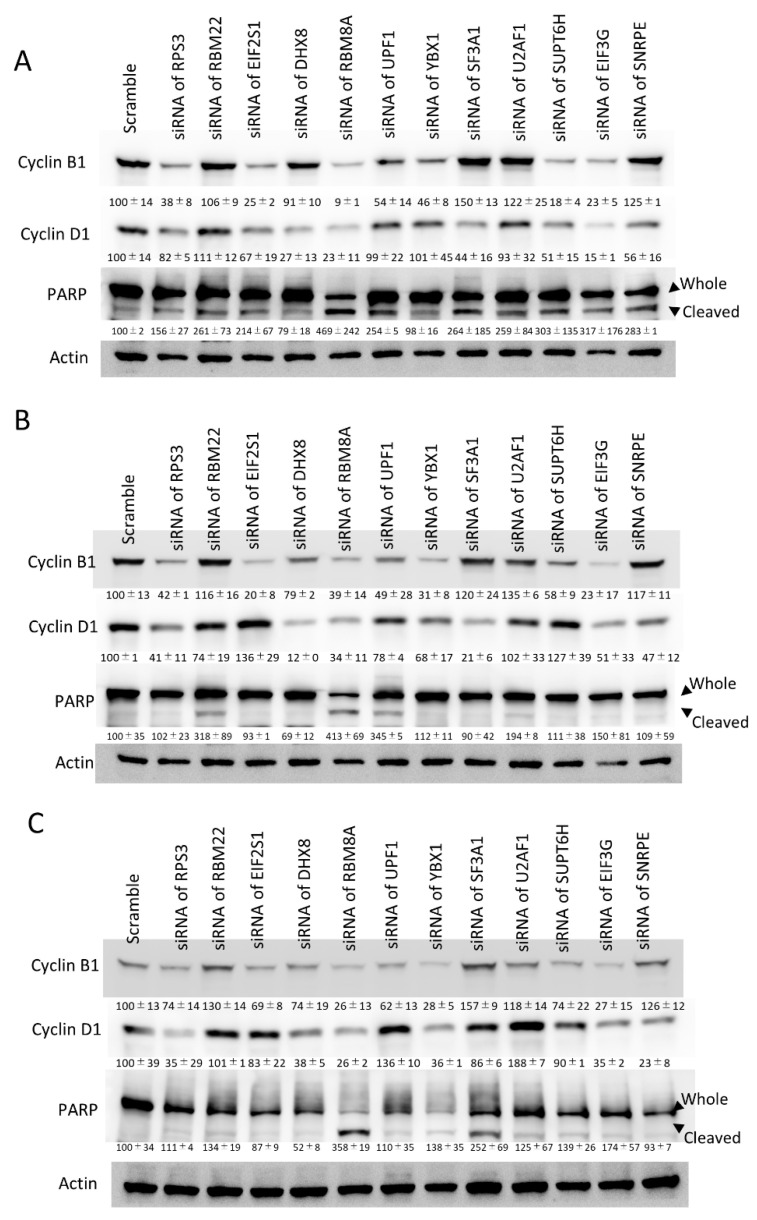
The aberrant expression of cell cycle and apoptosis-related molecules by the downregulation of RPS3, RBM22, EIF2S1, DHX8, RBM8A, UPF1, YBX1, SNRPE, SF3A1, U2AF1, SUPT6H, EIF3G in HCT116, SUIT2 and OE33 cells. A Western blotting analysis showed the expressional changes induced by the downregulation of cancer-related RBPs in HCT116 (**A**), SUIT2 (**B**) and OE33 (**C**) cells. The numbers of densitometry were described under each blot.

To confirm the growth suppression effect of these 12 RBPs, an MTT assay was performed. The growth of HCT116, SUIT2 and OE33 cells was significantly decreased in cells treated with siRNA of each RBP (Figure 4). To assess the influence of these 12 RBPs on the cell cycle and apoptosis, the protein expression of cyclin B1, cyclin D1 and cleaved PARP was examined by Western blotting. In HCT116 and SUIT-2 cells, the expression of cyclin B1 and/or cyclin D1 was decreased by the downregulation of these RBPs, with the exception of RBM22 and U2AF1 (Figure 5A,B). Interestingly, cleaved PARP was augmented by the downregulation of RBM22 and U2AF1, suggesting that RBM22 and U2AF1 inhibited apoptosis and promoted the tumor progression in colorectal cancer cells through cyclin B1 and/or cyclin D1-dissociated mechanisms. In OE33 cells, the expression of cyclin B1 and/or cyclin D1 was decreased by the downregulation of these RBPs, with the exception of RBM22 and U2AF1 (Figure 5C). Interestingly, the induction of cleaved PARP was not strongly induced by the downregulation of RBM22 and U2AF1, suggesting that some RBPs promoted tumor progression via a tissue-specific mechanisms.

### 3.4. Interactome Analysis of RBPs in Cancer Cells

**Figure 6:**
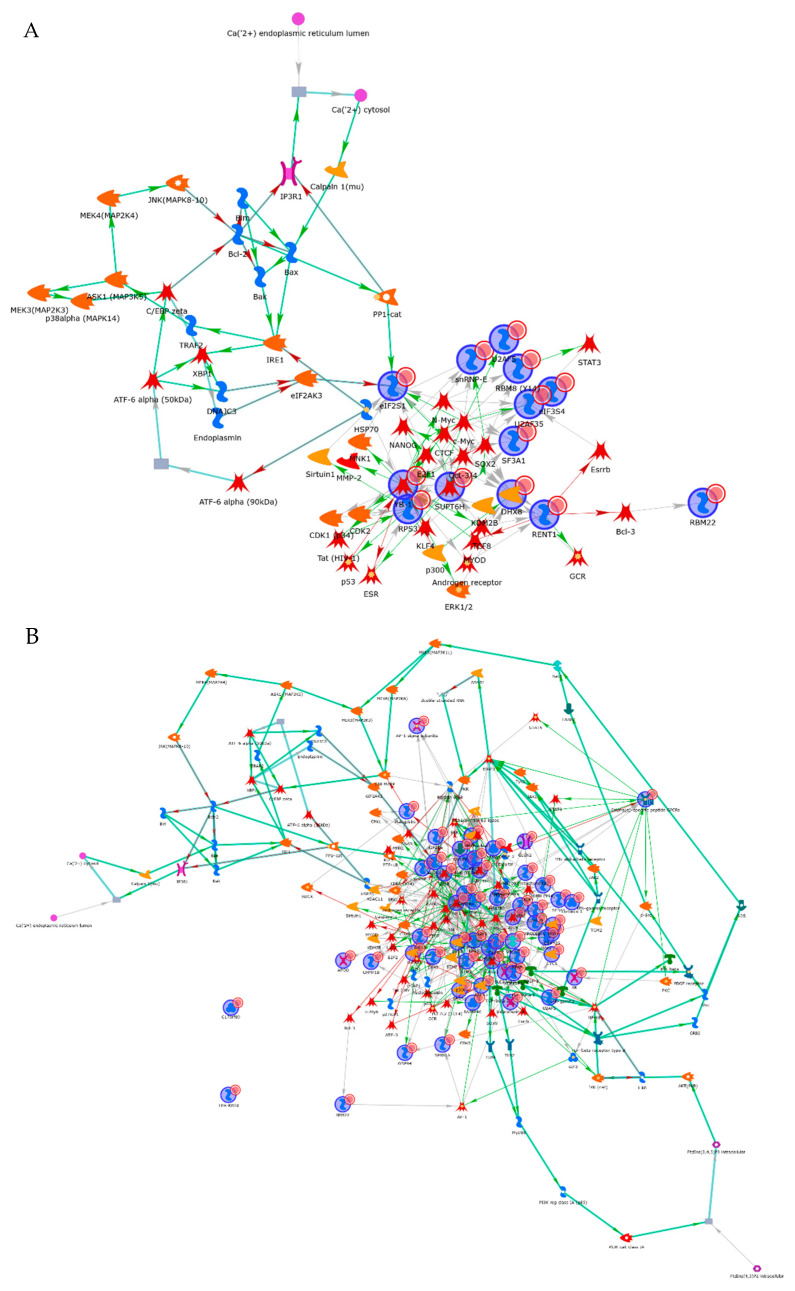
The network analysis of identified cancer-related mRNAs and aberrantly expressed mRNAs in cancer cells. The expression of identified cancer-related RBPs was regulated by the classical cell survival signal pathway (**A**). mRNAs that are overexpressed in cancer cells were shown to be regulated by the identified cancer-related RBPs in cancerous cells (**B**).

To assess the interaction network of these 12 RBPs, an interactome analysis was performed using the MetaCore software program. These RBPs constructed a cell survival network mediated by classical pathways, including ERK, p53 and Myc signal transduction (Figure 6A). To evaluate the interaction between identified RBPs and mRNAs, the gene set of abnormally augmented mRNAs was selected by a cDNA array analysis. A total of 41 mRNAs were aberrantly overexpressed in all cancer cell lines compared with non-cancerous cell lines (Table S18). To assess the relationship between the 12 RBPs and 41 mRNAs, these gene lists were uploaded to the MetaCore software program, and a gene network analysis was performed. Among 12 the RBPs, 9 RBPs (RPS3, YBX1 (alternative name: YB-1), RBM8A (alternative name: RBM8(Y14)), RBM22, UPF1 (alternative name: RENT1), SNRPE, U2AF1 (alternative name: U2AF35), EIF3G (alternative name: eIF3), SUPT6H) comprised the cancer-associated network through the regulation of transcriptional factors that are key regulators of cancer cell growth, apoptosis or differentiation (Figure 6B and Table 4). These data indicated that the cancer cell survival signal is regulated by identified RBPs.

### 3.5. Identification of Organ-Specific RBPs Exhibiting the Tumor-Promotive Function

To identify organ-specific tumor-promotive RBPs, we compared the tumor-promotive function of RBPs among HCT116, SUIT-2 and OE33 cells. Our functional assay showed that 18 RBPs (ACO1, ALKBH8, CELF2, EIF4H, KRR1, NOL8, PABPN1, PDCD11, RAVER1, RBM20, RBM35A, RNF113A, ROD1, SAMD4A, SFRS7, SLBP, SNRPB2, SRSF5), 24 RBPs (CPSF4L, ELAVL4, PABPN1L, PPRC1, RBM12, RBM18, RBM19, RBM27, RBM43, RBM44, RBM45, RBM4B, RBM7, RBMY1A1, RC3H1, SFRS6, TDRD10, THUMPD2, TUT1, ZC3H14, ZC3H3, ZC3H6, ZC3HAV1, ZFP36) and 35 RBPs (BICC1, HNRNPH1, LARP1B, LARP4B, LEMD3, LSM3, LSM4, MKRN1, MOV10L1, NIP7, NOVA1, NUPL2, PABPC1, PABPC1L2A, PABPC1L2B, PABPC5, PARP10, PCBP2, PUM1, RBM14, RBM23, RBM28, RBM39, RBM4, RBMS2, SFRS2, SFSWAP, SNRPN, SRBD1, ZC3H12B, ZC3H4, ZGPAT, ZNF74, ZRANB2, ZRSR2) were specifically promoted in colorectal, pancreatic and esophageal cancer cells, respectively (Table S19), suggesting that the tumor-promotive functions of some RBPs are dependent on the origins of cancers.

### 3.6. Identification of Tumor Growth Associated RBPs without Genetic Mutations in Gastrointestinal Cancer Cells

To elucidate whether or not cancer cell proliferation was associated with genetic mutations of RBPs, information on genetic mutations of each cell line was obtained from COSMIC and compared with the findings of our functional growth inhibition analysis using an siRNA library. A total of 144 mutations of RBPs in HCT116, 6 in SW480, 27 in SUIT-2, 12 in PANC-1, 13 in OE33 and 42 in KYSE-70 were identified in the COSMIC database (Tables S20–S25). Furthermore, 59, 3, 86, 19, 99 and 17 tumor-promoting RBPs identified by a functional analysis were not associated with genetic mutations in the HCT116, SW480, SUIT-2, PANC-1, OE33 or KYSE-70 lines, respectively (Tables S26–S31). Among these, 42, 3, 59, 9, 60 and 9 RBPs were not overexpressed in the HCT116, SW480, SUIT-2, PANC-1, OE33 and KYSE-70 lines, respectively (Table 5).

## 4. Discussion

We showed that a global functional analysis was a useful and critical strategy for identifying therapeutic targets in cancer cells. In the present study, we focused on RBPs because of their tumor-associated functions and identified candidate driver RBPs in cancer cell growth. Interestingly, we identified 12 tumor growth-promotive RBPs with an expression that did not change in cancer cells. We found that the genetic mutations registered in the COSMIC database do not necessarily reflect the molecular function of RBPs. The molecular group of RBPs without expressional or mutational changes as well as those with expressional or mutational changes appear to be viable therapeutic targets for cancer treatment.

In the present study, the list of RBPs that showed an altered expression in cancer cells was compared to the list of tumor-promotive RBPs identified by our functional analysis using the siRNA library. RBPs for which the growth-promotive function was associated with overexpression represented a relatively minor group in comparison to the RBPs without any significant expressional changes or non-tumor-promotive RBPs with significant expressional changes. These data indicated that the expressional changes in cancer cells include numerous secondary changes induced by alterations of tumor driver genes and that these changes are not suitable for therapeutic targets, even if they are useful as molecular markers. These results strongly suggested that an in silico database analysis to identify therapeutic targets based on significant expressional changes may miss many tumor growth-associated genes.

Likewise, we identified common tumor-promotive RBPs by a functional screening assay that could not be detected based on the expression data in colorectal, esophageal and pancreatic cancer cells. We clarified that 12 RBPs (RPS3, RBM22, EIF2S1, DHX8, RBM8A, UPF1, YBX1, SNRPE, SF3A1, U2AF1, SUPT6H, EIF3G) promoted tumor cell growth without expressional changes in all six cancer cell lines. Notably, strong growth inhibition was not detected in any non-cancerous cells (HCEC-1CT, HPPEC and Het1A) treated with the siRNA of nine RBPs (DHX8, EIF3G, RBM22, SF3A1, SNRPE, SUPT6H, U2AF1, UPF1, YBX1), suggesting that these RBPs possess a tumor-specific growth effect and are therefore attractive therapeutic targets in digestive cancer. Interestingly, our functional analysis revealed that most RBPs were associated with cancer cell progression, irrespective of the expressional changes; thus, global RBP inhibition may be a multipotent therapeutic target in cancer.

SNRPE is a component of spliceosomes that regulates pre-mRNA splicing through the formation of U snRNA assembly [18]. Our functional assay showed that SNRPE had a strong growth-promotive function in all cancer cell lines (HCT116, SW480, SUIT2, PANC1, OE33 and KYSE70 cells), but not in non-cancerous cells (HCEC-1CT, HPPEC and Het1A), suggesting that SNRPE is a strong candidate as a therapeutic target for tumor therapy. The mechanism underlying tumor promotion involves the mediation of the cell cycle promotion rather than mitosis, as cyclin D1, but not cyclin B1, was decreased in HCT116, SUIT-2 and OE33 cells by the downregulation of SNRPE. Importantly, our cDNA analysis showed that SNRPE is not increased in cancerous cells, indicating that the posttranscriptional modification of SNRPE is involved in tumor cell growth. 

SF3A1 is considered to play an important role in spliceosome assembly and mRNA splicing [19]. Our functional assay showed that SF3A1 strongly promoted the cell growth in cancer cells (HCT116, SUIT-2, OE33 and KYSE-70 cells) specifically, indicating that SF3A1 is a viable therapeutic target for tumor therapy. Interestingly, Western blotting revealed a decrease in cyclin D1 in colorectal cancer cells (HCT116 cells) and pancreatic cancer cells (SUIT2 cells) but not esophageal cancer cells (OE33 cells) by the downregulation of SF3A1. Likewise, the cleavage of PARP, a marker of apoptosis, by the downregulation of SF3A1 was detected in HCT116 and OE33 cells but not in SUIT2 cells. These findings suggest that the mechanism underlying cancer progression involving the mediation of SF3A1 has cancer tissue specificity. Our cDNA array analysis showed that SF3A1 is not dramatically increased in cancer cells, suggesting that post-translational modification rather than the augmentation of SF3A1 is also associated with cancer cell progression.

SUPT6H plays a key role in the regulation of transcription elongation and mRNA processing [20]. Our functional study showed the tumor proliferative function in gastrointestinal cancer cells (HCT116, SUIT-2, OE33 and KYSE70 cells), suggesting that the tumor-progressive property of SUPT6H is regulated by the posttranslational regulation. SUPT6H is associated with DNA repair in glioblastoma cancer stem-like cells (GSCs) [21] and estrogen-regulated transcription in breast cancer cells [22]. In the present study, Western blotting revealed a decrease in cyclin D1 and cyclin B1 and the augmentation of cleaved PARP in HCT116 cells in association with the downregulation of SUPT6H. In contrast, the decrease in cyclin D1 and cleavage of PARP were not detected in SUIT2 or OE33 cells, suggesting that the tumor-promotive mechanisms of SUPT6H also depend on the cancer cell type. A cDNA array analysis revealed no significant changes in the expression of SUPT6H in gastrointestinal cancer cells, suggesting that posttranscriptional regulation is a key step in cancer progression. The posttranscriptional mechanism of SUPT6H should be clarified to facilitate its use as a cancer therapeutic target in the future.

DHX8, EIF3G, RBM22, U2AF1 UPF1 and YBX-1 are also candidates therapeutic targets for cancer therapy because the tumor cell progression was strongly inhibited by the downregulation of these RBPs in cancer cells, including HCT116, SUIT2 and OE33 cells, but not in non-cancerous cells. DHX8 regulates the splicing, including p21 [23], and is re-quired for the release of mature mRNA from the spliceosome [24]. Western blotting showed that cyclin D1 is strongly decreased by the downregulation of DHX8, suggesting that DHX8 promoted the cell cycle in gastrointestinal cancer cells. EIF3G, which initiates mRNA translation, is required for protein synthesis [25]. Western blotting showed that the expression of cyclin B1 and cyclin D1 was decreased by the downregulation of EIF3G, suggesting that the cell cycle and mitotic events are controlled by EIF3G in cancer cells. A cDNA array analysis revealed no significant changes in the expression of EIF3G in gastrointestinal cancer cells. Thus, EIF3G is an attractive therapeutic target for gastrointestinal cancer. RBM22 regulates pre-mRNA splicing as a component of the activated spliceosome [26]. Western blotting showed that cleaved PARP was increased by the downregulation of RBM22 in HCT116 and SUIT-2 cells but not OE33 cells, suggesting that RBM22 inhibited apoptosis, thereby promoting the progression of colorectal and pancreatic cancer. No significant changes in the expression of cyclin B1, cyclin D1 or cleaved PARP were detected in RBM22-downregulated OE33 cells, suggesting that in esophageal cancer cells, the cancer cell growth was regulated by another mechanism that was not mediating the expressional changes in cyclin B1, cyclin D1 or cleaved PARP. U2AF1 is associated with the recognition of the 3′ splice site during pre-mRNA splicing [27]. Western blotting showed the augmentation of cleaved PARP in HCT116 and SUIT2 cells, suggesting that U2AF1 exerted an anti-apoptotic function in colorectal and pancreatic cancer cells. The cleavage of PARP was not detected, while the accumulation of cyclin D1 was observed in OE33 cells. These data suggest that the tumor-promotive mechanism that mediates U2AF1 also differs according to the type of cancer. 

UPF1 is an RNA-dependent helicase, and ATPase is required for nonsense-mediated decay (NMD) of mRNAs containing premature stop codons [28]. Western blotting showed that cyclin B1 was decreased by the downregulation of UPF1 in HCT116, SUIT-2 and OE33 cells, suggesting that abnormal cell division occurred in UPF1-downregulated cancer cells. Interestingly, Li et al. showed that the overexpression of UPF1 increased nonsense-mediated mRNA decay (NMD), resulting in decreased gastric cancer cell progression [29]. These conflicting functions of UPF1 show that UPF1 is an essential molecule for monitoring the RNA quality of cancer cells. Therefore, a cytotoxic effect on cancer cells was detected in our functional study using siRNA of UPF1 and in previous studies using the overexpression strategy.

YBX-1 can bind with DNA as well as RNA and is involved in various processes, including translational repression, RNA stabilization, mRNA splicing, DNA repair and transcription regulation [30,31,32]. In the present study, Western blotting showed that cyclin B1 was attenuated by the downregulation of YBX-1 in HCT116, SUIT2 and OE33 cells, suggesting that YBX-1 also regulates the cell mitotic events in cancer cells. It was previously reported that YBX1 is phosphorylated and activates Akt signal transduction in non-small-cell lung cancer cells [33]. Thus, we hypothesized that the tumor-promotive activities of YBX-1, such as phosphorylation, would be maintained and that the expression of YBX-1 mRNA would be attenuated in cancer cell lines.

Our functional assay showed that growth suppression was induced in non-cancerous cells by the downregulation of three RBPs (RPS3, EIF2S1, RBM8A), suggesting that these molecules have pivotal functions in proliferation and apoptosis in non-cancerous cells as well as cancer cells. RPS3, which is a component of the 40S small ribosomal subunit and associated with translation, controls the inflammatory response through the transactivation and proteasomal degradation of NFκB [34]. EIF2S1 catalyzes the first regulated step of the initiation of protein synthesis, promoting the binding of the initiator tRNA to 40S ribosomal sub-units. Eif2s1(tm1RjK) mice, in which Ser51 of eukaryotic initiation factor 2 subunit alpha (eIF2α, the homolog of EIF2S1) is mutated, do not survive after birth due to hypoglycemia associated with defective gluconeogenesis caused by a homozygous mutation [35]. RBM8A is a key component of the exon junction complex (EJC) and contributes to neurodevelopment through the regulation of p53 [36]. These reports indicate that RPS3, EIF2S1 and RBM8A contribute to the maintenance of non-cancerous cell homeostasis. Therefore, there is a possibility of adverse effects when RPS3, EIF2S1 and/or RBM8A are targeted in cancer therapy.

Our functional assessment of RBP combined with a bioinformatics analysis showed that the classical cell survival pathways, including Akt and p53, were essential for the exertion of the tumor-promotive functions by the RBPs identified in this study. Likewise, this interactome analysis identified the comprehensive interactions between these RBPs and abnormally expressed mRNAs in cancer cells. As each RBP is associated with numerus mRNAs and each mRNA is controlled by numerous RBPs, interactions between RBPs and mRNA are quite complicated. Our functional assessment with an interactome analysis is a powerful tool for identifying pivotal mechanisms regarding the expression/stabilization of mRNAs by RBPs. Therefore, a functional assessment is a useful strategy for investigating unknown mechanisms of tumor progression, as well for identifying therapeutic targets for tumor therapy.

Our microarray analysis using epithelial-like tumor cell lines showed significant differences in 12 RBPs between cancer and non-cancerous cells, whereas our in silico database analysis showed that two RBPs (RPS3, SNRPE), eight RBPs (RBM22, EIF2S1, RBM8A, UPF1, YBX1, SNRPE, DHX8, SF3A1) and two RBPs (SNRPE, SF3A1) were upregulated in colorectal, pancreatic and esophageal cancer tissue, respectively (Figure S1). Various types of cells, including epithelial cells, immune cells and fibroblasts, make up the tumor microenvironment in cancerous tissues and are involved in the development of tumor conditions associated with progression, including angiogenesis. In this study, we focused on epithelial-like tumor cells and assessed the influence of RBPs on growth. These RBPs may be induced in the tumor microenvironment to construct cells other than epithelial-like tumor cells and support tumor progression.

In this study, we assessed the growth promotive function of RBPs in cancers with three different origins and different clinical processes and identified the tumor type-specific RBPs in colorectal, pancreatic and esophageal cancer cells. Our functional assay revealed that 18 RBPs in colorectal cancer cells, 24 in pancreatic cancer cells and 35 in esophageal cancer cells supported tumor cell growth in specific tissue. These data mean that the contribution of RNA regulation mediating RBPs to cancer progression differs according to the type of cancer and that these tissue-specific RBPs may be suitable as therapeutic targets for tumor therapy in each organ.

Genetic mutations are well known to induce functional abnormalities of cancer-associated proteins, such as EGFR, KRAS and APC, resulting in cancer progression [15,37,38,39]. Thus, the selection of molecular targets based on genetic mutations has been considered an effective strategy in the development of cancer treatment. We examined the mutations of RBPs registered in the COSMIC database and compared them with the data of our functional growth inhibition analysis using an siRNA library. Interestingly, we found that numerous tumor-promoting RBPs identified by functional analyses were not associated with genetic mutations in cancer cells, showing that a functional analysis could identify many tumor-promoting RBPs, even those that could not be identified by mutational analyses.

As described above, our functional screening strategy can be used to identify additional novel cancer driver genes by targeting different molecular groups, such as transcriptional factors and growth factors. While this study used a procedure for screening cancer cell growth, similar strategies using migration or engraftment assays can identify key molecules for invasion and metastasis in cancers. Likewise, this strategy can be used to identify therapeutic targets of various disease, such as autoimmune diseases and neurodegenerative diseases.

## 5. Conclusions

In conclusion, we identified 12 RBPs (RPS3, RBM22, EIF2S1, DHX8, RBM8A, UPF1, YBX1, SNRPE, SF3A1, U2AF1, SUPT6H, EIF3G) with strong tumor proliferative effects without any marked changes in expression in colorectal, pancreatic and esophageal cancer using the functional assessment of RBPs with an siRNA library focused on cancer growth change. We also identified cancer driver genes from RBPs through the combination of a functional screening analysis and a cancer mutational database. These RBPs with or without changes in their expression or gene mutations are expected to be candidates for the development of novel molecular-targeting therapy.

## Figures and Tables

**Table 1:** Characteristics of each cell line.

Name	Characteristics	Organs	Morphology	Supplier
HCT116	Colorectal cancer cells	Colon	Epithelial	American Type Culture Collection (ATCC)
SW480	Colorectal cancer cells	Colon	Epithelial	American Type Culture Collection (ATCC)
HCEC-1CT	Immortalized colorectal epithelial cells	Colon	Epithelial	Evercyte
SUIT2	Pancreatic cancer cells	Pancreas	Epithelial	Health Science Research Resources Bank
PANC-1	Pancreatic cancer cells	Pancreas	Epithelial	American Type Culture Collection (ATCC)
HPPEC	Primary pancreatic epithelial cells	Pancreas	Epithelial	Cell Biologics, Inc.
OE33	Esophageal cancer cells	Esophagus	Epithelial	European Collection of Authenticated Cell Cultures (ECACC)
KYSE70	Esophageal cancer cells	Esophagus	Epithelial	European Collection of Authenticated Cell Cultures (ECACC)
Het-1A	Immortalized esophageal epithelial cells	Esophagus	Epithelial	American Type Culture Collection (ATCC)

**Table 2:** The growth suppression effect and expressional changes.

Gene Symbol	siRNA ID	Growth Inhibition Effect						Array ID	Fold Change					
		HCT 116	SW 480	SUIT -2	PANC -1	OE 33	KYSE 70		HCT 116	SW 480	SUIT -2	PANC −1	OE 33	KYSE 70
*DHX8*	s4018	94.6%	10.7%	64.5%	39.5%	88.8%	20.8%	TC1700012263.hg.1	1.28	1.10	−1.02	−2.79	−1.43	−2.39
	s4017	91.5%	41.8%	58.4%	61.3%	109.1%	47.3%							
	s4016	80.8%	10.3%	60.7%	53.5%	75.8%	36.9%							
*EIF2S1*	s4557	92.0%	10.5%	79.1%	24.9%	63.0%	42.5%	TC1400007494.hg.1	1.29	1.39	−1.45	1.01	−1.04	1.24
	s4556	90.1%	4.2%	80.1%	30.4%	65.1%	42.5%							
	s4555	87.0%	31.0%	92.7%	44.1%	66.7%	48.9%							
*EIF3G*	s225017	89.6%	26.7%	88.0%	72.9%	84.2%	67.6%	TC1900009608.hg.1	1.34	1.01	−1.80	−2.10	−1.42	1.02
	s16505	88.8%	39.2%	84.5%	46.7%	77.1%	70.8%							
	s16504	83.2%	47.8%	90.7%	67.3%	85.1%	79.0%							
*RBM22*	s31272	95.3%	44.3%	54.7%	49.5%	61.3%	53.3%	TC0500012485.hg.1	1.71	1.78	1.05	1.77	−1.82	−1.66
	s31273	54.6%	15.5%	53.9%	43.9%	55.4%	28.3%							
	s31274	50.8%	1.0%	41.0%	14.7%	37.8%	29.6%							
*RBM8A*	s532199	121.7%	116.8%	112.3%	75.7%	106.9%	49.2%	TC0100018477.hg.1	1.48	1.22	1.55	1.81	−1.08	−1.11
	s532200	113.3%	53.9%	108.5%	74.2%	115.4%	36.7%							
	s19292	112.1%	73.5%	97.3%	62.7%	112.4%	64.9%							
*RPS3*	s12255	96.2%	48.9%	88.6%	60.3%	79.8%	71.9%	TC1100008462.hg.1	1.26	1.43	1.15	−1.21	1.08	−1.34
	s12257	91.3%	37.6%	83.5%	80.1%	78.2%	85.0%							
	s12256	90.5%	66.0%	91.8%	52.6%	74.5%	85.3%							
*SF3A1*	s20116	105.1%	38.7%	105.7%	57.3%	89.0%	60.7%	TC2200008434.hg.1	1.62	1.26	1.99	1.88	−1.33	−1.00
	s20118	96.7%	11.5%	79.0%	59.2%	63.7%	54.1%							
	s20117	88.1%	49.7%	85.1%	43.5%	79.3%	51.1%							
*SNRPE*	s13238	102.4%	56.8%	89.4%	77.9%	75.8%	74.4%	TC0100011276.hg.1	1.85	2.00	1.42	1.36	1.57	1.52
	s13239	92.4%	27.6%	75.3%	55.8%	81.8%	64.3%							
*SUPT6H*	s13636	87.3%	24.6%	70.8%	55.5%	88.3%	71.7%	TC1700007381.hg.1	−1.05	−1.32	1.07	−1.05	1.18	−2.53
	s13634	84.9%	64.5%	64.2%	48.9%	76.9%	54.5%							
	s13635	71.7%	40.4%	62.9%	48.6%	61.6%	72.7%							
*U2AF1*	s14555	60.2%	15.4%	77.6%	51.2%	68.8%	30.7%	TC2100008286.hg.1	1.92	2.82	1.80	1.28	1.21	3.73
	s14553	57.8%	38.4%	92.4%	30.1%	85.3%	20.6%	TC2100007492.hg.1	1.85	2.68	1.73	1.67	1.34	2.85
	s14554	50.3%	14.0%	104.1%	18.6%	68.2%	10.9%							
*UPF1*	s11926	83.0%	11.9%	98.0%	32.4%	64.6%	43.3%	TC1900007410.hg.1	1.41	1.04	−1.36	3.15	−1.08	−1.49
	s11928	54.9%	7.4%	90.3%	44.1%	80.6%	34.9%							
	s11927	10.6%	−0.2%	−13.6%	32.3%	34.2%	9.8%							
*YBX1*	s9733	99.2%	14.0%	78.9%	28.9%	100.2%	10.8%	TC0100008026.hg.1	1.25	1.42	1.12	1.03	−1.29	1.11
	s9731	60.8%	18.9%	68.5%	75.6%	97.5%	36.2%	TC0100008025.hg.1	1.09	1.32	−1.25	−1.14	−4.14	−3.16
	s9732	41.7%	15.7%	41.4%	19.9%	37.2%	14.1%							

**Table 3:** The growth inhibition effect in non-cancerous cells.

Gene Symbol	Growth Inhibition Effect		
	HCEC-1CT	HPPEC	Het1A
*DHX8*	27.0%	11.3%	−14.9%
*EIF2S1*	52.9%	21.8%	6.4%
*EIF3G*	20.8%	18.0%	19.9%
*RBM22*	11.0%	29.0%	−2.1%
*RBM8A*	53.4%	69.3%	−8.5%
*RPS3*	58.1%	46.2%	2.1%
*SF3A1*	24.9%	−6.7%	−10.6%
*SNRPE*	34.4%	37.8%	17.0%
*SUPT6H*	35.5%	22.8%	49.0%
*U2AF1*	8.4%	0.9%	39.8%
*UPF1*	31.9%	31.9%	17.0%
*YBX1*	29.9%	31.9%	−2.1%

**Table 4:** The relationship of identified RBPs and transcriptional factors.

From	To	Effect	Mechanism
Bcl-3	RBM22	Transcription regulation	Unspecified
c-Myc	RBM8 (Y14)	Transcription regulation	Unspecified
N-Myc	RBM8 (Y14)	Transcription regulation	Unspecified
SOX2	RBM8 (Y14)	Transcription regulation	Unspecified
Androgen receptor	RENT1	Transcription regulation	Unspecified
KDM2B	RENT1	Co-regulation of transcription	Unspecified
Oct-3/4	RENT1	Transcription regulation	Unspecified
TCF8	RENT1	Transcription regulation	Unspecified
Beta-catenin	snRNP-E	Co-regulation of transcription	Unspecified
c-Myc	snRNP-E	Transcription regulation	Unspecified
NANOG	snRNP-E	Transcription regulation	Unspecified
N-Myc	snRNP-E	Transcription regulation	Unspecified
SOX2	snRNP-E	Transcription regulation	Activation
SOX9	snRNP-E	Transcription regulation	Unspecified
CDK2	SUPT6H	Phosphorylation	Unspecified
c-Myc	SUPT6H	Transcription regulation	Unspecified
NANOG	SUPT6H	Transcription regulation	Unspecified
SOX2	SUPT6H	Transcription regulation	Unspecified
TCF8	SUPT6H	Transcription regulation	Unspecified
c-Myc	U2AF35	Transcription regulation	Activation
CTCF	U2AF35	Transcription regulation	Unspecified
Esrrb	U2AF35	Transcription regulation	Unspecified
N-Myc	U2AF35	Transcription regulation	Unspecified
Oct-3/4	U2AF35	Transcription regulation	Unspecified
STAT3	U2AF35	Transcription regulation	Unspecified
c-Myc	YB-1	Transcription regulation	Activation
E2F1	YB-1	Transcription regulation	Activation
ERK1/2	YB-1	Phosphorylation	Inhibition
ERK2 (MAPK1)	YB-1	Phosphorylation	Activation
HSP60	YB-1	Binding	Inhibition
HSP70	YB-1	Binding	Activation
MYOD	YB-1	Transcription regulation	Activation
NANOG	YB-1	Transcription regulation	Unspecified
Oct-3/4	YB-1	Transcription regulation	Unspecified
p53	YB-1	Binding	Inhibition
STAT1	YB-1	Transcription regulation	Unspecified
Ubiquitin	YB-1	Binding	Inhibition
YB-1	Androgen receptor	Transcription regulation	Unspecified
RENT1	ATF-3	Binding	Inhibition
RENT1	Bcl-3	Binding	Inhibition
YB-1	CDK1 (p34)	Transcription regulation	Unspecified
SUPT6H	CDK2	Co-regulation of transcription	Unspecified
YB-1	CDK2	Transcription regulation	Activation
RBM22	c-Fos	Co-regulation of transcription	Unspecified
YB-1	c-IAP1	Transcription regulation	Activation
eIF3	c-Jun	Binding	Activation
RENT1	c-Myb	Binding	Inhibition
SUPT6H	c-Myc	Co-regulation of transcription	Unspecified
YB-1	c-Myc	Binding	Activation
YB-1	CTCF	Binding	Activation
RPS3	E2F1	Binding	Activation
YB-1	E2F2	Transcription regulation	Activation
YB-1	ERK2 (MAPK1)	Binding	Activation
YB-1	ESR	Binding	Inhibition
SUPT6H	ESR1 (nuclear)	Binding	Activation
YB-1	ESR1 (nuclear)	Binding	Inhibition
RENT1	Esrrb	Binding	Inhibition
YB-1	EZH2	Transcription regulation	Unspecified
RENT1	GCR	Binding	Activation
YB-1	GRP78	Binding	Inhibition
YB-1	HDAC11	Transcription regulation	Unspecified
snRNP-E	HSPA4	Co-regulation of transcription	Unspecified
YB-1	KDM2B	Transcription regulation	Unspecified
RENT1	Keratin 8	Binding	Inhibition
YB-1	KLF4	Transcription regulation	Activation
YB-1	KLF5	Transcription regulation	Unspecified
YB-1	MMP-2	Transcription regulation	Activation
YB-1	MNK1	Transcription regulation	Activation
RENT1	MYOD	Ubiquitination	Inhibition
SUPT6H	NANOG	Co-regulation of transcription	Activation
YB-1	NANOG	Binding	Activation
RPS3	NF-kB1 (p50)	Binding	Activation
YB-1	N-Myc	Binding	Activation
YB-1	Nucleophosmin	Transcription regulation	Unspecified
SUPT6H	Oct-3/4	Co-regulation of transcription	Activation
YB-1	p300	Binding	Inhibition
RPS3	p53	Binding	Activation
YB-1	p53	Transcription regulation	Inhibition
YB-1	p63	Transcription regulation	Unspecified
YB-1	PDGF-B	Transcription regulation	Unspecified
SUPT6H	PR (nuclear)	Co-regulation of transcription	Activation
YB-1	PTP-1B	Transcription regulation	Activation
RPS3	RelA (p65 NF-kB subunit)	Binding	Activation
RPS3	Sirtuin1	Binding	Activation
YB-1	SNAIL1	Binding	Activation
snRNP-E	snRNP-F	Binding	Activation
SUPT6H	SOX2	Co-regulation of transcription	Activation
YB-1	SOX2	Transcription regulation	Inhibition
RBM8 (Y14)	STAT3	Binding	Activation
YB-1	Tat (HIV-1)	Binding	Activation
YB-1	TCF8	Transcription regulation	Activation
YB-1	TGF-beta 1	Binding	Inhibition
YB-1	TGF-beta 2	Transcription regulation	Unspecified

**Table 5:** The list of tumor-promotive RBPs whose expressional abnormality and mutation were not detected in cancer cells.

Cell Lines	Gene List					
HCT116	*CPEB4*	*MKI67IP*	*RPS3*	*SNRPB*	*YBX1*	
	*DHX8*	*NOL8*	*RRP7A*	*SNRPB2*	*ZC3H12C*	
	*EIF2S1*	*PABPN1*	*SAMD4A*	*SNRPC*		
	*EIF3G*	*PNO1*	*SF3A1*	*SNRPE*		
	*HNRNPA1L2*	*RAVER1*	*SF3B14*	*SRRM2*		
	*HNRNPAB*	*RBM22*	*SF3B4*	*SRSF5*		
	*HNRNPCL1*	*RBM8A*	*SFRS13A*	*SUPT6H*		
	*LSM2*	*RNF113A*	*SFRS3*	*TIA1*		
	*LSM5*	*RNPS1*	*SFRS7*	*TLR2*		
	*LSM8*	*ROD1*	*SLBP*	*U2AF1*		
SW480	*RBM8A*					
	*SF3B14*					
	*SNRPB*					
SUIT-2	*CHERP*	*LARP6*	*RBM11*	*RPS3*	*SNRPE*	*UPF3B*
	*CPSF4*	*LIN28B*	*RBM18*	*SF3A1*	*SNRPG*	*YBX1*
	*CPSF4L*	*LSM6*	*RBM22*	*SF3B14*	*SRRM2*	*YTHDC1*
	*DAZL*	*LSM8*	*RBM43*	*SFRS13A*	*SUPT6H*	*ZC3H12D*
	*DHX8*	*MKI67IP*	*RBM45*	*SFRS3*	*TDRD10*	*ZC3H15*
	*EIF2S1*	*NCL*	*RBM4B*	*SFRS6*	*TIA1*	*ZC3H18*
	*EIF3B*	*NONO*	*RBM7*	*SNRPC*	*TUT1*	*ZC3HAV1*
	*EIF3G*	*PABPN1L*	*RBM8A*	*SNRPD1*	*U2AF1*	*ZC3HAV1L*
	*HNRNPAB*	*POLR2G*	*RBMY1A1*	*SNRPD2*	*U2SURP*	*ZFP36L1*
	*HNRNPK*	*PUF60*	*RC3H1*	*SNRPD3*	*UPF1*	
PANC-1	*RBM8A*	*SF3B14*	*SNRPD3*			
	*RPS3*	*SNRPD1*	*SNRPE*			
	*SF3A1*	*SNRPD2*	*SNRPG*			
OE33	*CARHSP1*	*LARP1*	*PABPC1L2A*	*RBM8A*	*SRBD1*	*ZC3H18*
	*CHERP*	*LARP6*	*PABPC1L2B*	*RPS3*	*SUPT6H*	*ZC3H4*
	*CSDC2*	*LEMD3*	*PABPC5*	*RRP7A*	*U2AF1*	*ZC3H7B*
	*DAZL*	*LIN28B*	*PARP10*	*SF1*	*U2SURP*	*ZC3HAV1L*
	*DHX8*	*LSM2*	*PCBP2*	*SF3A1*	*UPF1*	*ZFP36L1*
	*EIF2S1*	*LSM3*	*PRPF3*	*SFSWAP*	*UPF3B*	*ZFP36L2*
	*HNRNPA1L2*	*MOV10L1*	*RBM14*	*SNRPD2*	*YTHDC1*	*ZGPAT*
	*HNRNPC*	*NONO*	*RBM22*	*SNRPD3*	*ZC3H11A*	*ZNF74*
	*HNRNPCL1*	*NUPL2*	*RBM25*	*SNRPE*	*ZC3H12B*	*ZRANB2*
	*HNRNPR*	*PABPC1*	*RBM39*	*SNRPG*	*ZC3H12D*	*ZRSR2*
KYSE-70	*EIF3G*	*PUF60*	*SNRPD2*			
	*LARP1*	*RPS3*	*SNRPE*			
	*NONO*	*SF3B4*	*SRRM1*			

## Data Availability

All datasets generated and/or analyzed during the current study are available from the corresponding author upon reasonable request.

## Supplementary Materials

The following are available online at https://www.mdpi.com/article/10.3390/cancers13133165/s1, Figure S1: An in silico database analysis of identified tumor-promotive RBPs. Figure S2: Unprocessed original scans of western blots. Table S1: The sequence of siRNAs of RBPs. Table S2: The list of RBPs whose growth inhibition effects is over 50% when siRNA of each gene was transfected to cancer cells. Table S3: Growth inhibition effect of HCT116. Table S4: Growth inhibition effect of SW480. Table S5: Growth inhibition effect of SUIT-2. Table S6: Growth inhibition effect of PANC-1. Table S7: Growth inhibition effect of OE33. Table S8: Growth inhibition effect of KYSE70. Table S9: Fold change of mRNA of RBPs (HCT116/HCEC-1CT). Table S10: Fold change of mRNA of RBPs (SW480/HCEC-1CT). Table S11: Fold change of mRNA of RBPs (SUIT-2/HPPEC). Table S12: Fold change of mRNA of RBPs (PANC-1/HPPEC). Table S13: Fold change of mRNA of RBPs (OE33/Het-1A). Table S14: Fold change of mRNA of RBPs (KYSE70/Het-1A). Table S15: The correlation of the tumor-promotive function and expression of RBPs. Table S16: The knockdown efficacy of siRNA in each cell line. Table S17: The list of RBPs that were not detected by the cDNA array analysis. Table S18: The list of aberrantly overexpressed mRNAs in cancer cells. Table S19: The list of organ-specific RBPs exhibiting the tumor promotive function. Table S20: Registered mutations in HCT116. Table S21: Registered mutations in SW480. Table S22: Registered mutations in SUIT-2. Table S23: Registered mutations in PANC-1. Table S24: Registered mutations in OE33. Table S25: Registered mutations in KYSE70. Table S26: Tumor-promotive RBPs without a mutation in HCT116. Table S27: Tumor-promotive RBPs without a mutation in SW480. Table S28: Tumor-promotive RBPs without a mutation in SUIT-2. Table S29: Tumor-promotive RBPs without a mutation in PANC-1. Table S30: Tumor-promotive RBPs without a mutation in OE33. Table S31: Tumor-promotive RBPs without a mutation in KYSE70.
