# Supplementary material for: The Identification of RNA-Binding Proteins Functionally Associated with Tumor Progression in Gastrointestinal Cancer
Source: Cancers (Basel). 2021 Jun 24;13(13):3165. doi: 10.3390/cancers13133165 (PMC8269357; doi:10.3390/cancers13133165)
Supplement: Supplementary file 1 [file cancers-13-03165-s001.zip › Supplementary information/Supplementary figures.pdf]

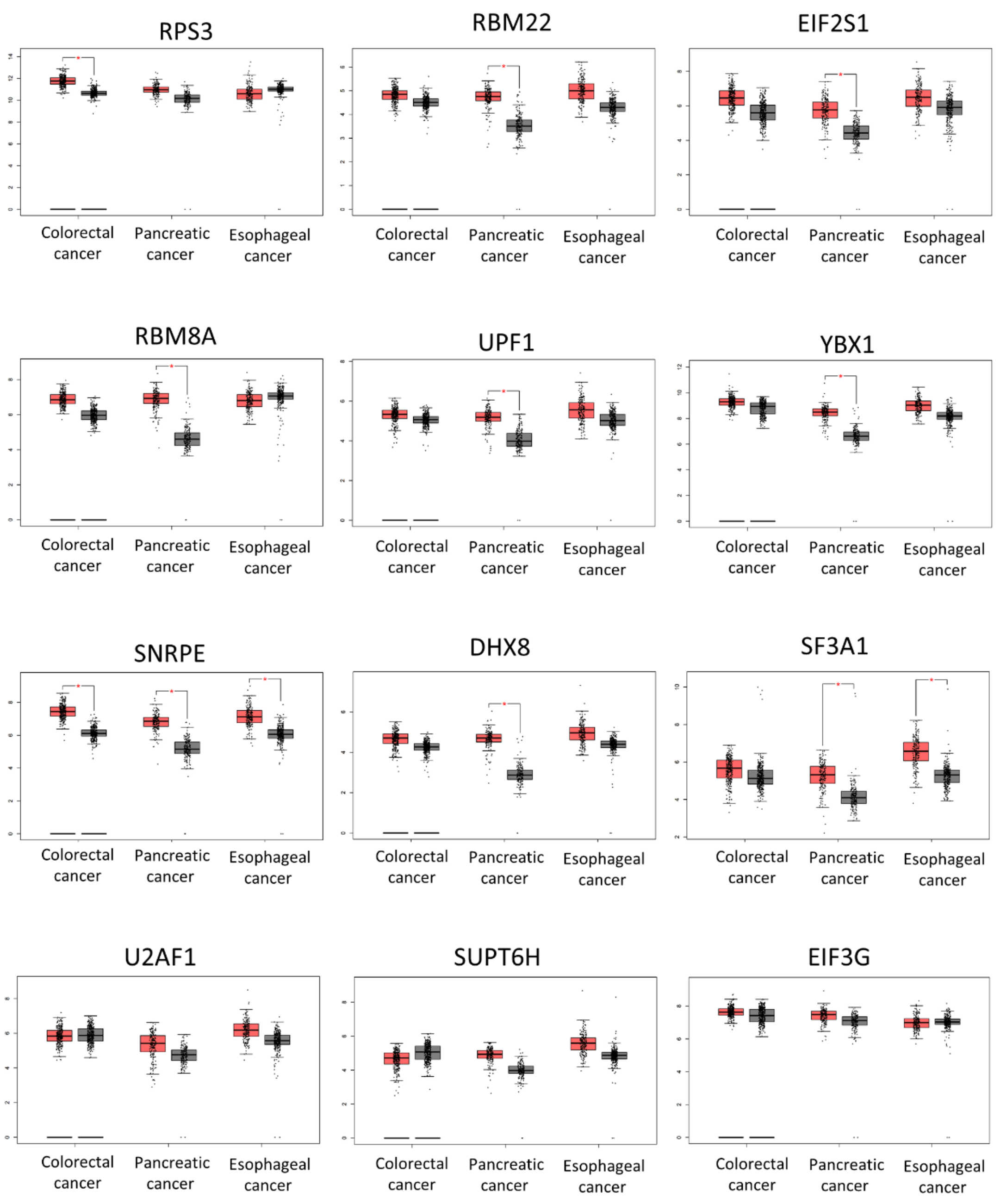

The number of sample (Colorectal cancer); n=275 (Tumor, red box), n=349 (Non-tumor, gray box)
The number of sample (Pancreatic cancer); n=179 (Tumor, red box), n=171 (Non-tumor, gray box)
The number of sample (Esophageal cancer); n=182 (Tumor, red box), n=286 (Non-tumor, gray box)

Supplementary Figure S1. In silico database analysis of identified tumor promotive RBPs
The expressional changes of cancer tissues of 12RBPs were compared to non-cancerous tissues. The data was downloaded from GEPIA (Gene Expression Profiling Interactive Analysis).

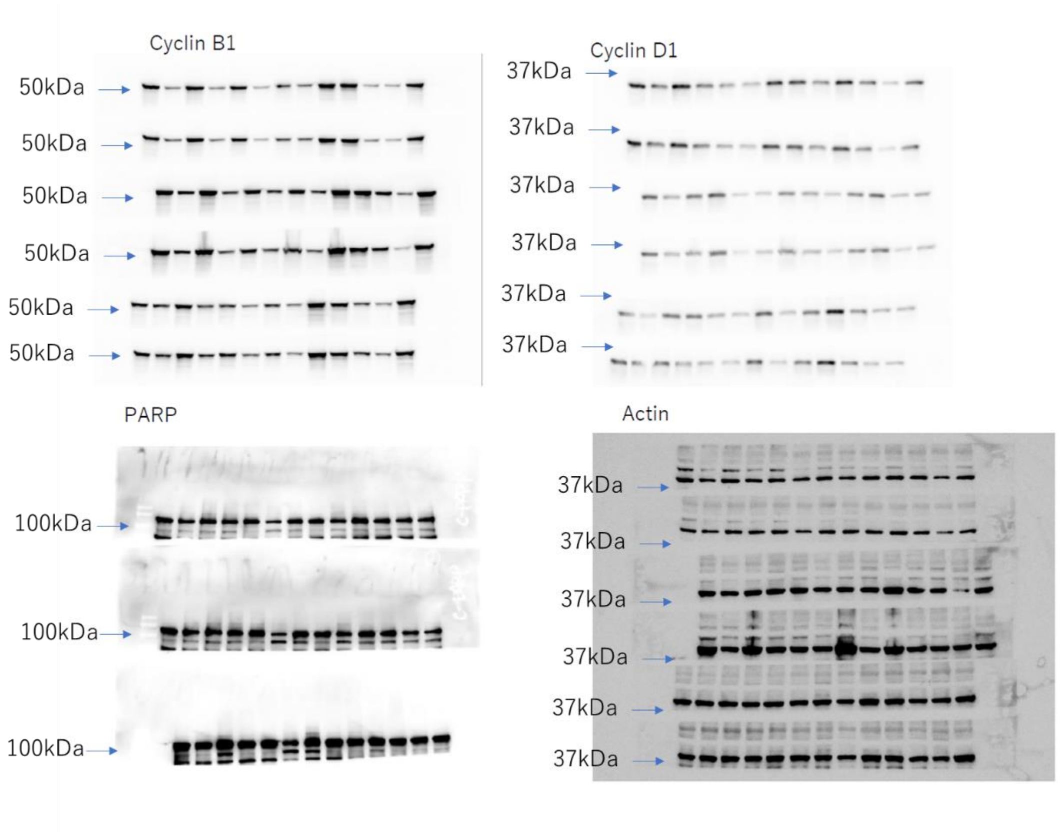

Supplemental Figure S2. Unprocessed original scans of western blots.
